# A molecularly enhanced proof of concept for targeting cocrystals at molecular scale in continuous pharmaceuticals cocrystallization

**DOI:** 10.1073/pnas.2114277119

**Published:** 2022-05-20

**Authors:** Milad Asgarpour Khansary, Saeed Shirazian, Gavin Walker

**Affiliations:** ^a^Confirm Smart Manufacturing, University of Limerick, Limerick, V94 C928 Ireland;; ^b^Department of Chemical Science, Bernal Institute, University of Limerick, Limerick, V94 T9PX Ireland;; ^c^Synthesis and Solid State Pharmaceutical Centre, Bernal Institute, University of Limerick, Limerick, V94 T9PX Ireland

**Keywords:** pharmaceuticals, cocrystallization, machine learning, molecular engineering

## Abstract

It is impossible to optimize a process for a target drug product with the desired profile without a proper understanding of the interplay among the material attributes, the process parameters, and the attributes of the drug product. There is a particular need to bridge the micro- and mesoscale events that occur during this process. Here, we propose а molecular engineering methodology for the continuous cocrystallization process, based on Raman spectra measured experimentally with a probe and from quantum mechanical calculations. Using molecular dynamics simulations, the theoretical Raman spectra were calculated from first principles for local mixture structures under an external shear force at various temperatures. A proof of concept is developed to build the process design space from the computed data. We show that the determined process design space provides valuable insight for optimizing the cocrystallization process at the nanoscale, where experimental measurements are difficult and/or inapplicable. The results suggest that our method may be used to target cocrystallization processes at the molecular scale for improved pharmaceutical synthesis.

## Significance

This contribution offers a proof of concept to make it possible to target a specific (co)crystal at molecular scale within a continuous process. The paper, while addressing a very important issue in public health, i.e., high-efficiency medicine production, also emphasizes the significance of computational material science and data science in generating proper understanding of a process and its optimal operating conditions.

Many drugs discovered in the past few decades are low in aqueous solubility (1), which is a very important indicator of bioavailability (2). After oral administration, drugs enter the stomach with an acidic aqueous environment, in which most active pharmaceutical ingredients show very poor solubility (3). Among many techniques developed to improve the solubility of drugs (4), cocrystal formation has become very common because it does not negatively impact the drug’s pharmacological properties (5). Besides better bioavailability, cocrystals have improved physicochemical properties including tabletability, stability, and permeability (6). The formed cocrystals usually consist of an active pharmaceutical ingredient and an approved component (known as a coformer) in stoichiometric ratio (7). There is a strong interest in cocrystals because they reduce the time and therefore the cost of drug development (8).

Among various developed cocrystallization processes (9, 10), solid-state synthesis is superior due to its high efficiency, low level of by-products, and no need for solvents (6). For continuous processing of pharmaceutical formulations using solid-state synthesis, twin-screw granulation is considered an excellent and promising technology (11, 12) that combines cocrystallization and granulation, with a short residence time and the possibility of conducting chemical reactions (13–15). Unfortunately, this technique is yet to be implemented on an industrial scale (16), essentially due to the lack of micro-/macroscopic insight into the compounds’ behavior and the proper process control strategies to optimize the formulations (17, 18).

Many researchers have investigated continuous cocrystallization via twin-screw granulators (19), as reviewed elsewhere (20–23). However, information from the experimental studies tends to be very limited and empirical because those studies often focus on analyzing the individual operating parameters in a trial-and-error approach (24, 25). Other problems with the experimental approach include material cost, implementation and reconfiguration of the twin-screw granulator, training human resources, and time consumption. On the other hand, the most sophisticated theoretical models currently available are practically top-to-bottom approaches and hence require the input of experimentally correlated parameters, such as particle size distribution (26, 27). Therefore, such models fail to bridge the gap between the micro- and mesoscales of continuous cocrystallization processing (28–30). Consequently, it is hardly possible to robustly synthesize an optimization procedure for a drug product to achieve the desired target product profile (31).

Solving the above problem requires reliable insight into the interplay of 1) the critical raw material attributes, 2) the critical process parameters, and 3) the drug product’s critical quality attributes. This in turn necessitates models that utilize a bottom-up approach (32, 33) [where the material properties are calculated from scratch using, e.g., density functional theory [DFT] and molecular dynamics [MD] (34)] to establish a process design space without prior experimental information. After producing this design space, a process optimization strategy can be synthesized for any specific operational parameters.

Here, we chose the cocrystallization process of ibuprofen (IBF) and nicotinamide (NCTA) for case study. Ibuprofen is a drug widely used to treat pain and fever (2, 35). Since it has very poor solubility in the stomach environment (3, 36), nicotinamide was used as a coformer for cocrystal formation (37, 38) via twin-screw granulator. The cocrystal structure is usually studied through spectroscopic techniques (39–41), mainly Raman spectroscopy (42). Examples include cocrystallization via twin-screw granulation (43) and in aqueous media during slurry conversion (44). Analysis of the Raman spectra can reveal whether interactions between the compounds are chemical or physical in nature (45). However, Raman spectroscopy in this context tends to be used as a tool for a product (end) quality check (46–48). In contrast, in the current study we used signals from the Raman spectrometer equipped on the twin-screw granulator to quantify interactions between compounds throughout the granulator. Depending on the identified interactions, the intensity of a specific interaction affecting the target cocrystal in formulation can be controlled, provided that one knows how to affect the stability and kinetics of that interaction through macroscopic processing parameters (such as the temperature and screw rotation speed) (49). This molecular-level information can bridge the gap between the micro- and mesoscales of continuous cocrystallization processing. Instead of exhaustive empirical experimentation, we determined the process design space from scratch through quantum mechanical methods, resulting in a protocol that requires no experiments, is generic, and can be applied to any system of interest. For the three considered processing parameters (temperature, shear rate as exerted by screw rotation speed, and residency time) in wide practical value ranges, we performed DFT and MD calculations to determine the possible interactions between ibuprofen and nicotinamide, as well as changes in their stability and kinetics. In particular, we calculated the Raman intensities as described by Porezag and Pederson (50). The computed Raman patterns were correlated with the three processing parameters using the proposed proof of concept, resulting in a process design space. This design space was compared for the target interaction, set as input, with the signals from the Raman spectrometer to estimate the proper temperature, shear rate, and residency time and therefore gauge the twin-screw granulator. The following sections discuss our developed approach and its implementation.

## Results and Discussion

Following the theoretical calculation, we performed a literature review to check the reliability/quality of the generated data. Our calculated solvation energies (ibuprofen: −60.18 kJ/mol; nicotinamide: −66.96 kJ/mol) were valid according to reported computations as well as experimentally measured solubilities (0.021 mg/mL and 24 mg/mL, respectively) (51–53). Our calculated melting temperatures (ibuprofen: 355.15 K; nicotinamide: 397.15 K, *mean values*) were also in agreement with the literature (ibuprofen: 353.15 K [54, 55] and nicotinamide: 398.5 K [56, 57], respectively). Other descriptors analyzed were the chemical potential (which is the negative of electronegativity), the highest occupied molecular orbital and lowest unoccupied molecular orbital, hardness, and electrophilicity index (58–62). Their values are provided in *SI Appendix*. While these descriptors are unique to each molecule, they do not provide a basis for designing the control mechanism. On the other hand, our computed Raman data of fingerprints as candidate descriptors agreed with the relevant works (63–72). The fingerprints’ structures and their corresponding normalized Raman intensities are summarized in *SI Appendix*, Fig. S1. The labels DI, DN, and CO represent the ibuprofen dimer, nicotinamide dimer (49, 73), and cocrystals, respectively.

**Fig. 1.**
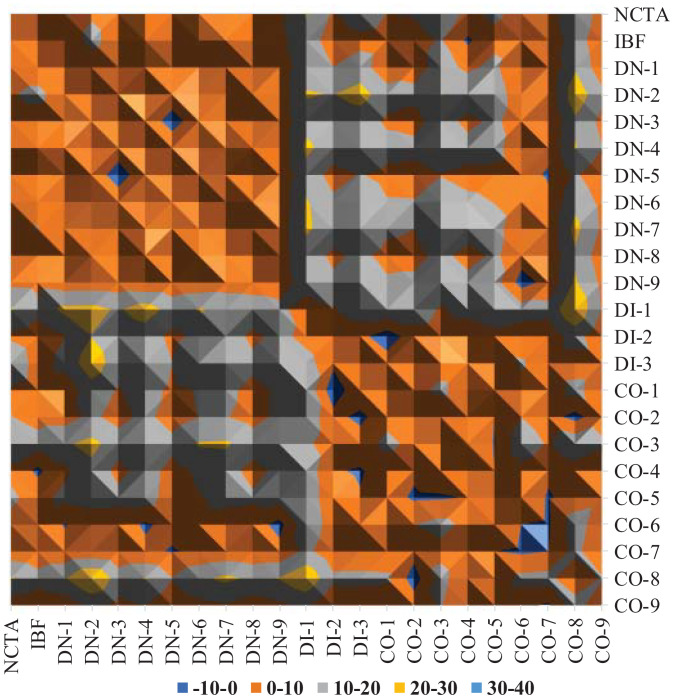
Interaction tendency of fingerprints based on the Flory–Huggins interaction parameter.

Initially, we employed the lattice solution theory of Flory–Huggins (74, 75) to examine the interactions among the fingerprints under at-rest (no shear) condition to check their possible coexistence (Fig. 1). Since most of the values were positive, these fingerprints were not expected to become mixed but rather to grow within their own phases. The dark-blue colored areas in Fig. 1 indicate the possibility of coexistence/mixing of the pair involved. Following our previous recommendation to mitigate dimer formation (49), during this initial examination we focused on the coexistence of cocrystals. There was coexistence compatibility between CO-6 and CO-7 but they had slow emergence kinetics, and the weak established electrostatic interaction made their presence rare. CO-2 showed coexistence compatibility with CO-8 and CO-5, but given the unfavorable solvation energy of CO-8, it was unstable and tended to dissociate. These findings suggest that CO-2 and CO-5 have a promising chance for growth.

**Fig. 2.**
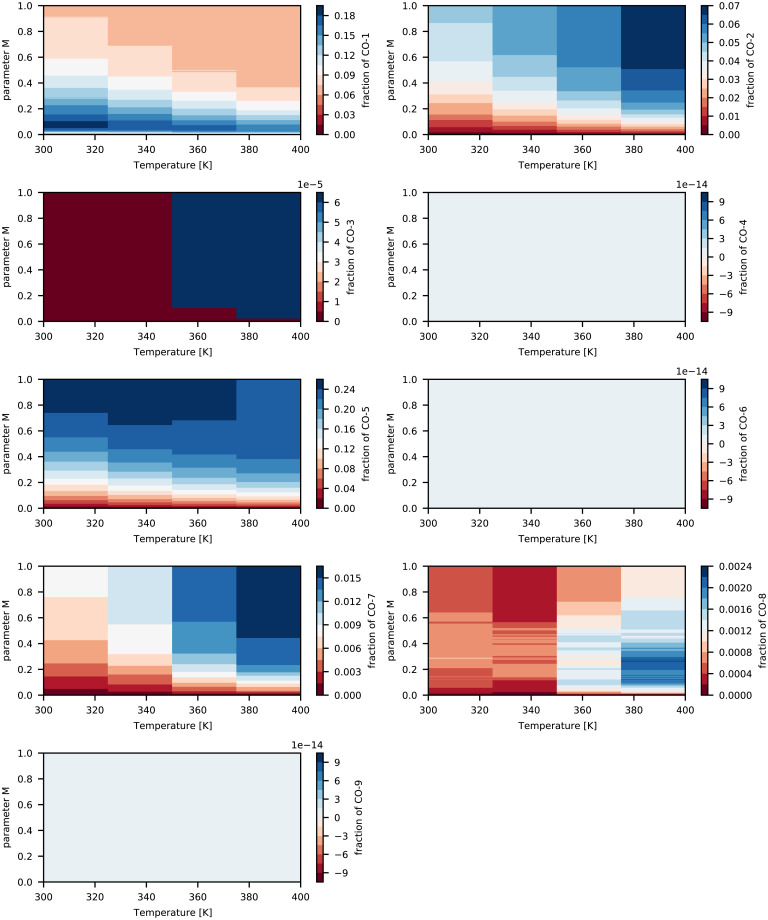
Computed design space {*T, τ, t, á_i_*} and parameter *M* for cocrystals (a blank image means a nearly zero value [<10^−5^] is calculated for particular fingerprint fraction).

**Fig. 3.**
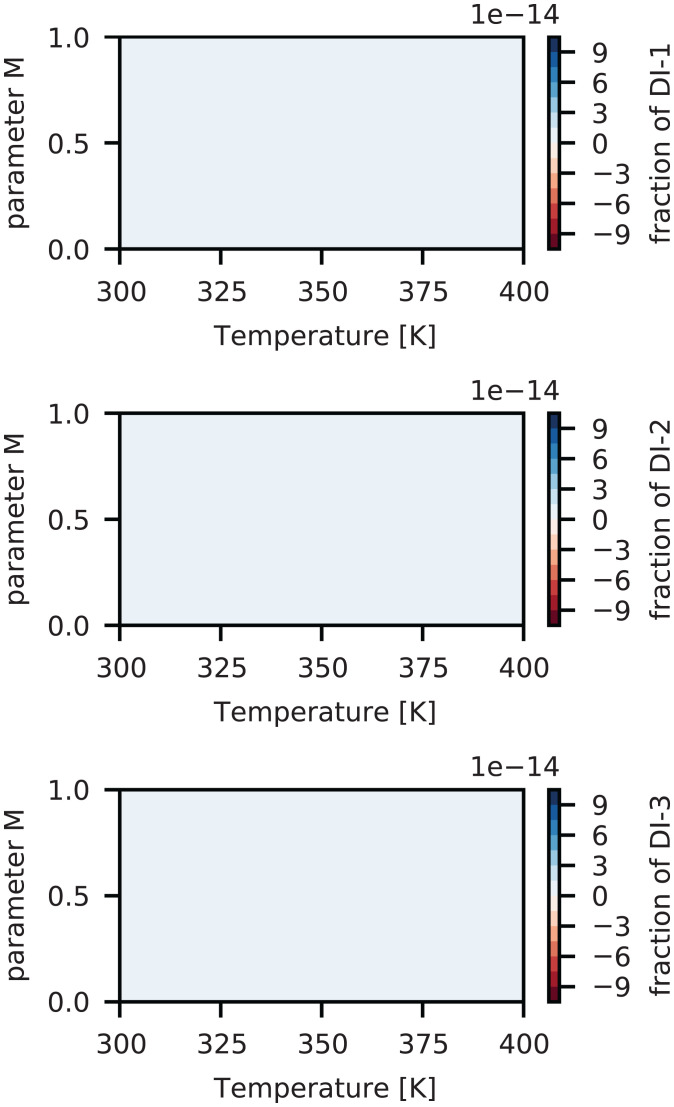
Computed design space {*T, τ, t, á_i_*} and parameter *M* for ibuprofen dimers (a blank image means a nearly zero value [<10^−5^] is calculated for particular fingerprint fraction).

**Fig. 4.**
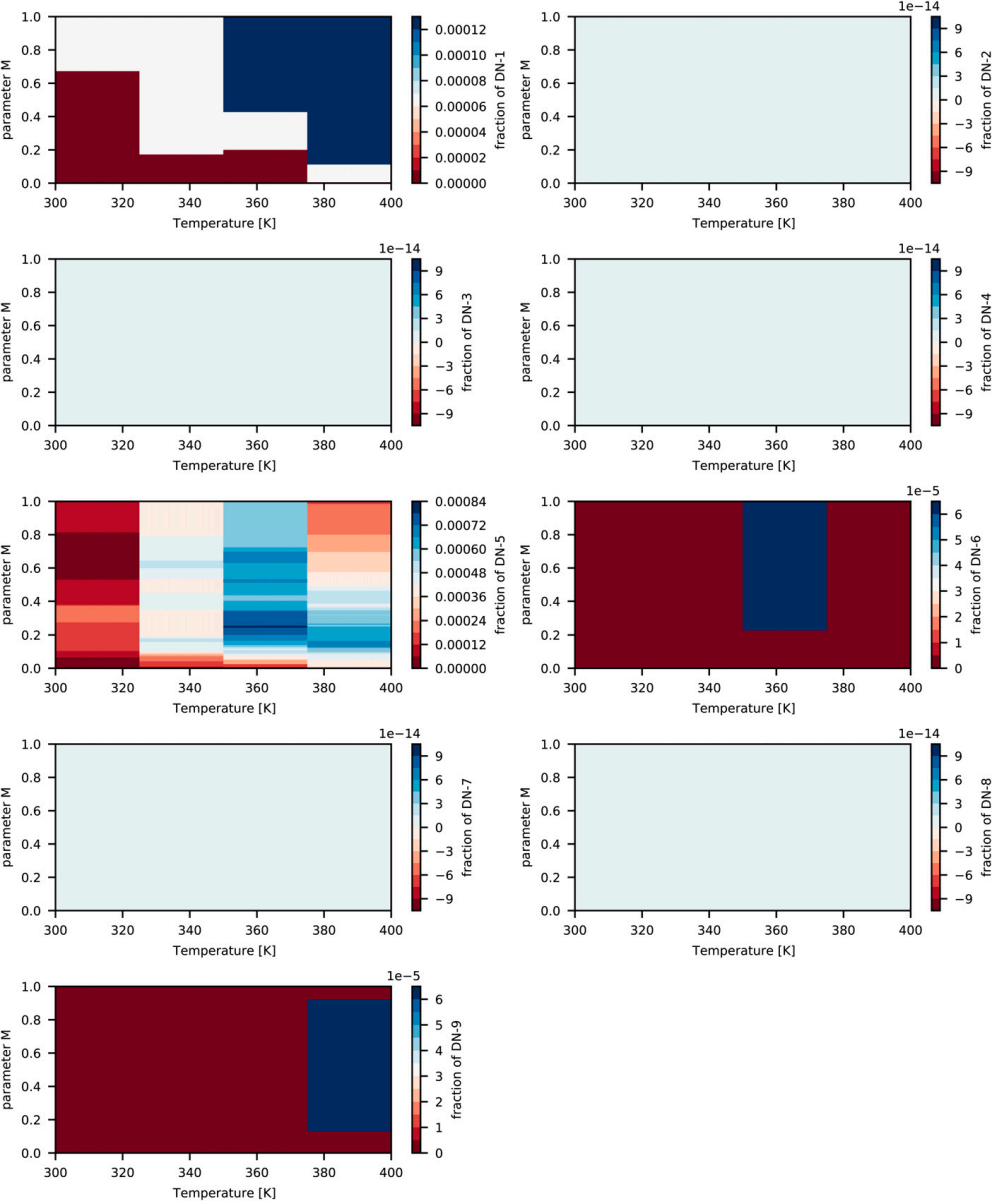
Computed design space {*T, τ, t, á_i_*} and parameter *M* for nicotinamide dimers (a blank image means a nearly zero value [<10^−5^] is calculated for particular fingerprint fraction).

**Fig. 5.**
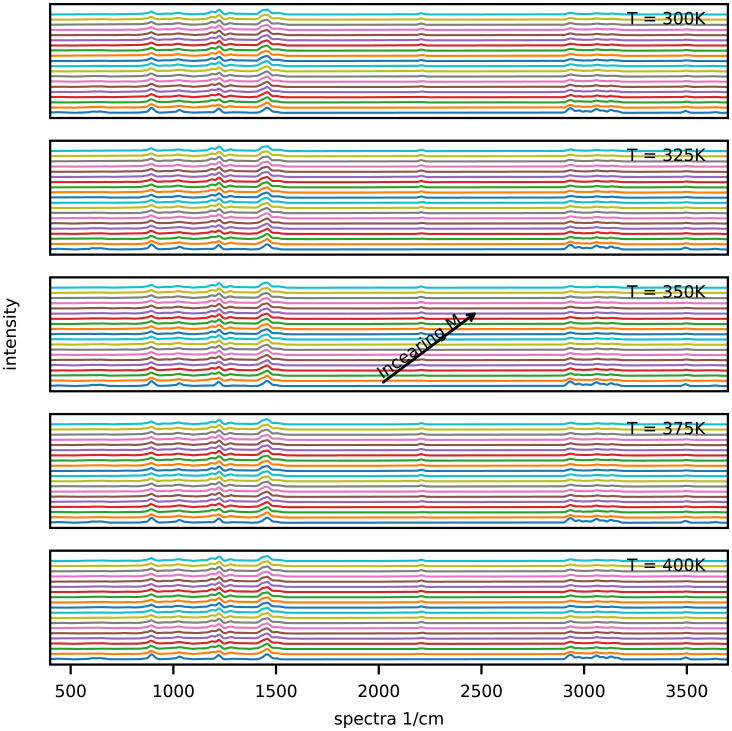
Computed Raman intensities design space and parameter *M*.

**Fig. 6.**
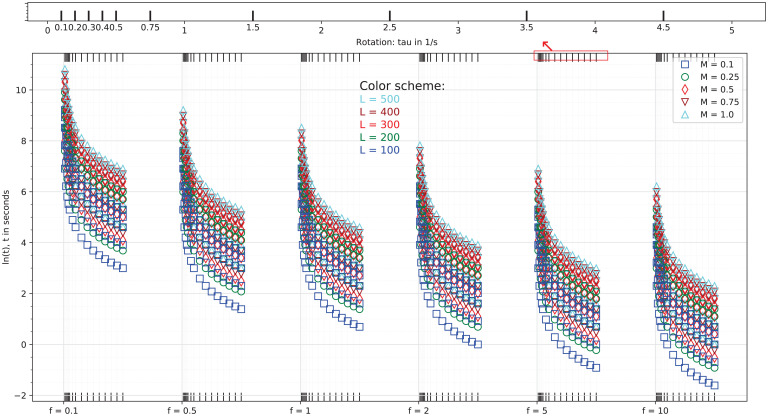
Computed design space in terms of parameter *M* (*L* is the length of the twin-screw granulator, *f* is the forward carrying of material per rotation [screw lead]).

The computed design space $\left\{T,\tau ,t,a_{i}^{\prime }\right\}$ is reported in Figs. 2–4 together with the associated computed normalized Raman intensities shown in Fig. 5 over the parameter *M*, which is the point of reference to start optimizing the operating specifications for a fingerprint of the interested fraction (as seen in Figs. 2–4). The computed design space in terms of *M* is shown in Fig. 6 for a nominal design specification of the twin-screw granulator. The value of *M* connects the two representations of design space. For example, by selecting *M* from Figs. 2–4 at the optimal temperature for the target fingerprint and determining the type of screw available (as represented by *f*), one can identify the optimal screw rotation from Fig. 6.

The distinct continuous growth and emergence of CO-5 can be seen from the peak within 2,000 to 2,500 cm^−1^ (see *SI Appendix*, Fig. S1 for each fingerprint's peak profile) in the computed Raman intensities as shown in Fig. 5 and fraction shown in Fig. 2 and Fig. 5. This fingerprint also appeared in the ibuprofen–nicotinamide cocrystal structure reported previously (76, 77). However, those analyses were restricted to the spectral range of 700 to 1,200 cm^−1^ to monitor the structure corresponding to the CO-2 fingerprint, and due to clipping the spectral range, those works missed to notice the associated peaks of CO-5. Applying the method of Emeis (78) to spectra within the 700 to 1,200 cm^−1^ range only, we can calculate that CO-2 has a maximum presence (i.e., > 80%), which is in agreement with the observation of the authors’ (76). Note that according to the design space (Figs. 2–4), almost no amount of CO-4, CO-6, CO-9, DI-1, DI-2, DI-3, DN-2, DN-3, DN-4, DN-7, or DN-8 could be expected at any values of *M* and temperature. The fraction of CO-1 appeared to reach a maximum of 20% for *M* < 0.08 but decreased to ∼7.5% as *M* approached 1. This finding was associated with the kinetics of CO-1 formation and its lower competitiveness against other fingerprints (73). Given the kinetic rates we previously reported (73), the favorable formation energy barrier associated with CO-1 formation allowed this step to start quickly. However, after more exchange of energy and mass during the process, the reverse process (CO-1 decomposition into ibuprofen and nicotinamide) became more favorable than its formation (73). The fraction of CO-2 increased systematically with *M* and temperature, reaching a maximum of 7% at the extreme boundary. The emergence of CO-3 could be safely ignored since its fraction was found to be very negligible. Meanwhile, CO-3 showed strong responses to *M* and temperature, jumping from 10^−3^% at the lower boundary to 6 × 10^−3^% at the upper boundary. The fraction of CO-7 systematically increased with *M* and temperature, reaching a maximum of 1.8% at the upper boundaries. That of CO-8 showed a strong sensitivity to temperature, jumping from 8 × 10^−2^% at the lowest temperature to 24 × 10^−2^% at the highest. At all temperatures, CO-8 initially increased with *M* and then decreased. This finding was associated with the formation and decomposition processes of CO-8, despite their relative rank in competitiveness against other molecular interactions. While the formation of CO-8 is favorable, its decomposition process requires energy built up within the system (73). A systematic increase was seen in the fraction of DN-1 according to *M* and the temperature (Fig. 3), jumping from 6 × 10^−3^% at the lowest temperature to 12 × 10^−3^% at the highest. Nevertheless, the presence of DN-1 could be safely ignored because of its very negligible fractions. Given the kinetics of DN-5 (73), the initial increase of its fraction with *M* and a subsequent decrease could be realized in a straightforward manner. The associated formation and decomposition processes have very similar kinetic rates, but the latter requires an energy input. Therefore, as the process progresses, the role of decomposition becomes more important, especially at elevated temperatures as seen in the fraction map. DN-6 seemed to be present in the ibuprofen–nicotinamide cocrystal structure reported previously (76, 77). The small fraction of DN-6 (maximum: ∼6 × 10^−3^% at 360 K) suggested the relative strength of CO-5 sharing the same nicotinamide molecule with DN-6. Unless the temperature exceeded 380 K, the fraction of DN-9 remained near zero, with the possibility of reaching at most 6 × 10^−3^% at some *M* values and disappearing as *M* approached 1. The DN-9 fraction remained near zero because it is formed at a similar rate as its decomposition to nicotinamide. Its emergence at 0.16 < *M* < 0.95 and disappearance as *M* approached 1 can be linked to the availability of additional nicotinamide due to the response of CO-8 to *M* and temperature.

The optimal condition for maximum cocrystal formation (primarily CO-5 and CO-2, with the other fingerprints in only trace amounts) is 340 K < *T* < 350 K and 0.4 < *M* < 0.55. Considering the design specification of the twin-screw granulator used here, the value of either the screw rotation or the lead should be determined based on the *M* value. This design space can be used as a controller to manipulate the operating parameters in real time, mainly the temperature and screw rotation speed. In such a scenario, we would solve Eq. **3** for the probe-measured Raman intensities as vector *R*, resulting in the real-time calculation of the fraction of fingerprints. The calculated fractions would be compared against the design space of Figs. 2–4, which would act as a decision tree for the controller to alter the screw rotation speed or temperature.

**Fig. 7.**
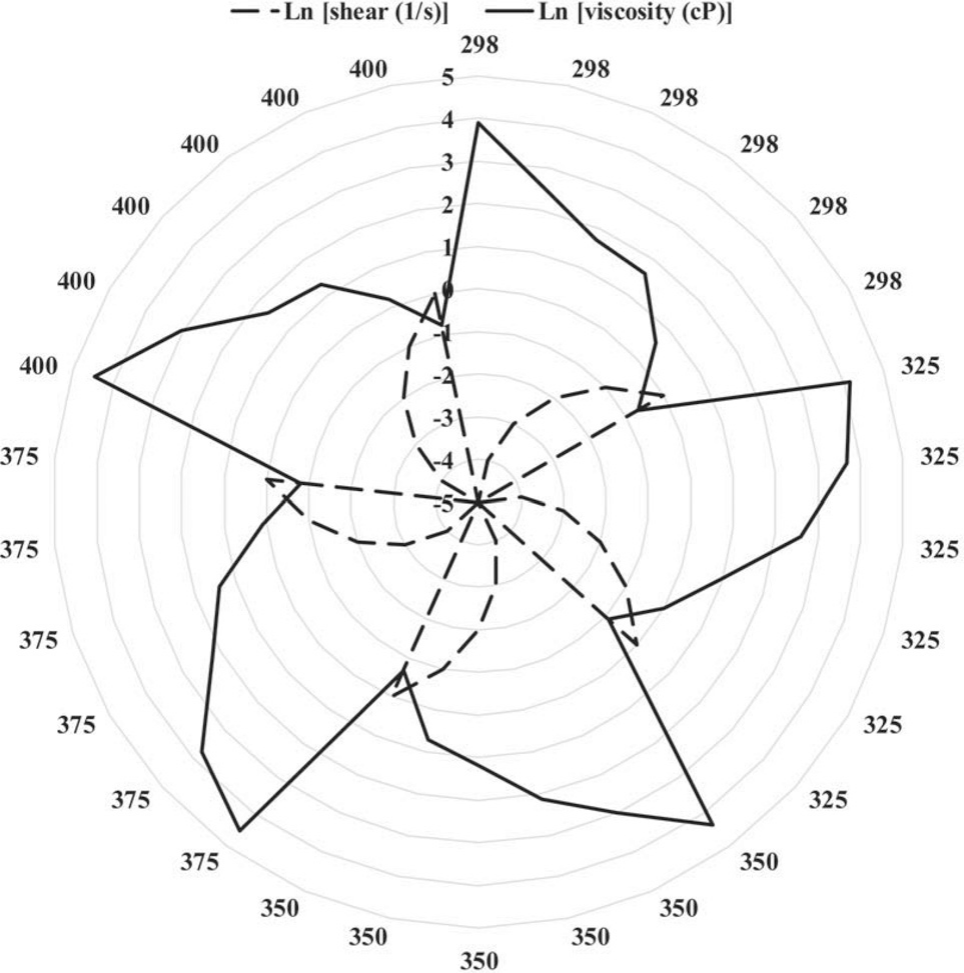
Variation in viscosity (cP) due to shear (s^–1^) over the temperature range of 298 to 400 K in logarithm (Ln) scale.

In practical applications, the twin-screw granulator is exposed to the ambient air without good thermal insulation, leading to heat exchange and thermal loss. In addition, the control and manipulation of temperature depend on the thermal response of the material used in manufacturing the twin-screw granulator. Thus, we believe more focus should be placed on the screw rotation speed as the control parameter, after setting the temperature within the optimal range. There may also be concerns about the reliability of screw rotation speed because of the flowability of the mixture along the twin-screw granulator. Indeed, we have noticed that the flowability of mixture varies along the twin-screw granulator (37). Over the temperature range of 298 to 400 K, we calculated the dynamic viscosity (in cP). The results were averaged over all data points and are reported in Fig. 7. At any temperature, the viscosity decreased with an increasing shear rate, reflecting a pseudoplastic behavior (non-Newtonian behavior at lower shear rates and Newtonian behavior at higher shear rates). At a higher shear rate, the molecules started to untangle from each other and align along the applied shear. Such molecular reordering resulted in a higher degree of order and consequently a lower overall stress. The general theory of Carreau (79) is handy for correlating the shear ($\dot{\gamma }$) with the viscosity (*μ*) as $\frac{\mu -\mu_{\infty }}{\mu_{0}-\mu_{\infty }}={\left[1+{\left(\lambda \dot{\gamma }\right)}^{\alpha }\right]}^{\frac{n-1}{\alpha }}$, where *μ_0_* and *μ_∞_* are the limiting viscosities at the low and high shear limits, respectively. *α* is usually chosen to be 2. *λ* and *n* are empirical constants and calculated to be 10^4^ and −0.35, respectively, by correlating all data points at all temperatures with *R*^2^ > 0.98. At intermediate and high shear rates, the Carreau equation is reduced to a power law in the form of $\mu =\mu_{0}{\left(\lambda \dot{\gamma }\right)}^{n-1}$, whereas in the low shear rate regime it is reduced to $\mu =\mu_{0}$. At the intersection of these two regimes located at $\dot{\gamma }=\frac{1}{\lambda }$, the viscosity and shear rate are the same. It can be seen from Fig. 7 that increasing the temperature decreased the viscosity due to reduced friction between the molecules. It is possible to correlate the viscosity variations with the temperature (Τ) at low shear rates by employing an Arrhenius-type equation of $\mu =A\times \exp\left[\frac{E}{RT}\right]$, where *R* is the universal gas constant and *A* and *E* are empirical constants (80) calculated to be A = 610.75 and $\frac{E}{R}$ = −736.64. In a regime where the viscosity is more sensitive to temperature, i.e., *T* > 35 °C, the following refined form of the aforementioned equation should be used: $\frac{\mu }{\mu_{0}}=\exp\left[E\left[\frac{1}{T}-\frac{1}{T_{0}}\right]\right]$. Therefore, we conclude that there is no need to worry about the reliability of the screw rotation speed.

## Concluding Remarks

We present a computational approach to build the process design space for cocrystallization in twin-screw granulators for process optimization and engineering. Based on our DFT data, we devised a proof of concept to extract the representative fractions of various fingerprints (molecular interactions) from the computed Raman intensities. When we employed the Raman data measured through a probe installed on the twin-screw granulator, the generated design created a control mechanism to manipulate the process parameters and improve the production of target cocrystal(s). The constructed design space allowed us to easily identify:

(1) The optimal parameters to run the twin-screw granulator for maximizing the production of ibuprofen–nicotinamide cocrystals without requiring trial-and-error experimentation. According to Figs. 2–4, the optimal condition is 340 K < *T* < 350 K and 0.4 < *M* < 0.55 for maximizing CO-5 and CO-2 with trace amounts of other fingerprints. To be more specific, knowing *M* allows the straightforward determination of either the screw rotation or *f* for the design specification of the twin-screw granulator, using Fig. 6.
(2) The gauging/adaptation procedure for real-time correction of the temperature and screw rotation speed. By solving Eq. **3** for the Raman intensities measured using a probe as the vector *R* in real time, the fraction of fingerprints was calculated. These calculated fractions were compared against the design space of Figs. 2–4, which acted as the decision tree of a controller to alter the screw speed or temperature.

## Computational Details

### Overview.

**Fig. 8.**
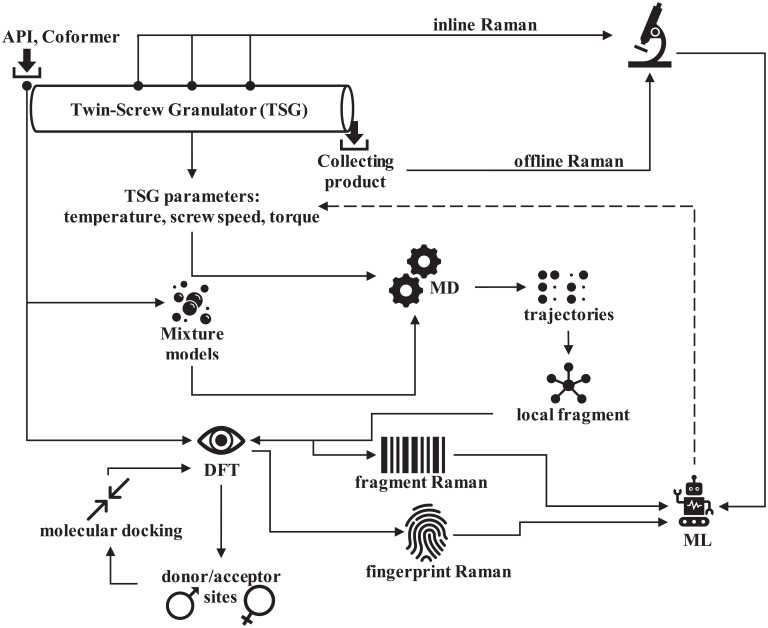
Schematic overview of the developed computational framework (API: active pharmaceutical ingredients, ML: machine learning, DFT: density functional theory, and MD: molecular dynamics).

The developed computational framework consists of two main layers: the molecular modeling (MM) layer and the proof of concept for machine learning (ML) layer. The MM layer uses DFT and MD calculations to generate molecular-level information about interactions among ibuprofen and nicotinamide as well as their variation under various operating conditions of the twin-screw granulator (i.e., in wide ranges of temperature, external shear force, and residency time). The ML layer is designed to create a computational design space by recognizing patterns among the operating conditions of the twin-screw granulator and the features of molecular interactions, to synthesize the relevant information for developing operational control strategies. We considered the Raman spectra as the feature of molecular interactions (descriptors). Analytical studies frequently use Raman spectroscopy as a tool (81) because it can show whether the interactions between compounds are chemical or physical in nature (45). The experimental Raman spectra are usually measured offline on samples collected at the end of the twin-screw granulator (44). However, it is also possible to collect these spectra inline, when the formation is passing through the twin-screw granulator, by using probes installed on top at different locations (43). By comparing the computed Raman data to the experimental measurements, we determined the types of possible molecular interactions and groups, which are detailed in the next paragraphs and shown in Fig. 8.

### Molecular Modeling Layer.

In this layer, first we used DFT calculations to identify the donor–acceptor sites on each molecule because molecules interact through these sites to form new phases/structures (82). In addition, DFT calculations generated the quantum data required for calculating the proper physicochemical descriptors of each molecule (83). The molecular structures of ibuprofen and nicotinamide were retrieved from the 69th reference database made available by the National Institute of Standards and Technology (USA). The molecular structures were optimized by employing the generalized gradient approximations with Perdew-Burke-Ernzerhof functional (84) including implicit solvent (85) as described by the conductor-like screening model (86). To control the convergence behavior for enhanced self-consistent field calculation (87, 88), thermal smearing (89) was also applied with a double numerical basis including the d-polarization function (90) level of theory. The double numerical basis with d-polarization incorporates diffuse functions (90) for the proper treatment of long-range effects, which were not negligible here. The convergence tolerances were 2.0 × 10^−5^ kcal/mol in energy, 10^−3^ kcal/mol/Å in force, maximum iterations of 10^4^, and displacement of 10^−5^ Å. The reasons for choosing this functional and these criteria were discussed in our previous work (49). We calculated surface electrostatic charges (91) using the Hirshfeld partitioning scheme (92). The sigma surface charge densities were calculated as introduced by Klamt and Schüürmann (93) and revisited elsewhere (94). We used electrostatic potential charges and sigma surface charge densities to identify the surface donor-acceptor sites on both ibuprofen and nicotinamide molecules (82, 95). The Raman spectra were calculated as described by Porezag and Pederson (50). We ignored the spectra below 400 cm^−1^ as those high frequencies are associated with phonons (69), and we strived to include frequencies as low as 3,750 cm^−1^ to account for solvent effects (49). In the current calculation of Raman data, the incident light had an intensity of 532 nm and the spectra were extracted at 20 cm^−1^ intervals, which corresponded to a laser power of 150 mW. These parameters matched the specifications of the Raman spectrometer available in our laboratory. Such spectral extraction resulted in 1,000 data points in each Raman intensity dataset. After we identified the surface donor/acceptor sites on ibuprofen and nicotinamide, all possible pairs for the two molecules were created by placing the donor site on one molecule in close contact with the acceptor site on an identical or different molecule following a molecular docking framework (49). This is because in a mixture, each molecule can undergo donor–acceptor exchange with another molecule of the same or different species. Therefore, in the binary mixture we had three macromolecular groups (pairs) formed through such molecular interactions: 1) ibuprofen dimers, 2) nicotinamide dimers, and 3) cocrystals of ibuprofen and nicotinamide. The dimers are formed due to donor–acceptor interactions between identical molecules. However, the more interesting (target) donor–acceptor exchanges occur when an ibuprofen molecule interacts with a nicotinamide molecule, representing plausible cocrystals of ibuprofen and nicotinamide. Here, a close contact is defined as a distance shorter than the van der Waals distance between the two molecules (49). For all these macromolecular groups (pairs), we performed the same DFT calculations as applied to isolated single molecules and calculated their Raman spectra as described by Porezag and Pederson (50). We referred to the Raman spectra of the macromolecular groups (pairs) as fingerprints while those of isolated single molecules were used for noise reduction in the data.

Next, to mimic the mixture conditions, we created molecular models of ibuprofen and nicotinamide in a 1:1 ratio. This ratio was based on the industrial practice for this specific system and the availability of literature data for further validation (37, 44, 55, 67, 68, 95, 96). The models contained 10 or 25 of each molecule under periodic boundary conditions so that we could account for possible effects of model size on the simulation results and enhance the reliability of our computation. For each mixture model, we performed structure relaxation using a reliable force field because of the high cost of DFT relaxation for such a large system of atoms. We used a refined version (97) of the consistent valence force field developed from ab initio energy surfaces (98). The convergence tolerances were 2.0 × 10^−5^ kcal/mol in energy, 10^−3^ kcal/mol/Å in force, maximum iterations of 10^4^, and displacement of 10^−5^ Å. To obtain the lowest energy structures, we tried to avoid local energy minima by performing 5 consecutive annealing (99, 100) cycles at up to 500 K for 75 ps for each molecular model of the mixture. The resulting structure was used for MD simulation under the constant number of molecules, pressure, and temperature (NPT) ensemble for a period of 1,000 ps, followed by another 1,000 ps dynamic run in the constant number of molecules, volume, and energy (NVE) ensemble (99, 100). This was done to apply the temperature effects at each desired operating temperature of the twin-screw granulator: 298, 325, 350, 375, and 400 K. This temperature range spans from room temperature to the melting point of the coformer (nicotinamide), i.e., the allowable operating temperatures for this system (49). We should emphasize that under the NPT and NVE ensembles, we were practically minimizing the Gibbs free energy and entropy, respectively (101). In these dynamic calculations, we used the velocity Verlet algorithm to integrate Newton’s equation of motion employing the Berendsen thermostat (102). These dynamic runs were repeated 10 times for each molecular model of the mixture to cancel out the random effects.

The final optimized and equilibrated structures at each temperature were used for MD simulation under external shear forces to investigate molecular reorientation and shear-induced molecular interactions (103–105). Shear rates of 1, 0.1, 0.01, 0.001, 0.0001, and 0.00001 ps^−1^ were applied to each mixture model at the upper facet under the NPT ensemble for a period of 1,000 ps (106–108). Trajectories were extracted every 0.5 ps. For each local mixture structure, the Raman spectra were calculated as described by Porezag and Pederson (50).

### Proof of Concept for ML Layer.

In the ML layer, we first normalized all Raman intensities as $\bar{x}=\left[x\;-\;\min\right]/\left[\max\;-\;\min\right]$, where *x* is the Raman intensity and *min* and *max* are the minimum and maximum intensities in each dataset, respectively. $0\leq \bar{x}\leq 1$ is the normalized Raman intensity. Note that each dataset contained 1,000 intensity data points. We correlated the normalized Raman spectra of the local mixture structure with those of the fingerprints at each environmental condition: the temperature, the shear rate, and the corresponding time stamp in trajectory. We employed the polynomial theory of complex systems (109) to generate the main kernel function in the development of correlations. This theory states that if the dependent variable *y* is determined by *N* independent variables $x_{1},x_{2},\ldots ,x_{N}$ according to an unknown functional $y=f\left(x_{1},x_{2},\ldots ,x_{N}\right)$, then it is possible to find an approximate functional form ($\bar{f}$) that represents the dependency and reproduces the dependent variable with an error of $E=y-\bar{y}$, where $\bar{y}$ is the reproduced (approximated) dependent variable. $\bar{f}$ can be expressed in the form of a Volterra functional series (109) as given in Eq. **1**, where $a_{0}$, $a_{i}$, $a_{ij}$, $a_{\mathrm{ijk}}$, $a_{\mathrm{ijkl}}$, and $a_{\mathrm{ijklm}}$ are constant coefficients.

[1] $$\begin{array}{l} \bar{y}=a_{0}+\sum_{i=1}^{N}a_{i}x_{i}+\sum_{i=1}^{N}\sum_{j=1}^{N}a_{ij}x_{i}x_{j}+\sum_{i=1}^{N}\sum_{j=1}^{N}\sum_{k=1}^{N}a_{\mathrm{ijk}}x_{i}x_{j}x_{k}\\ +\sum_{i=1}^{N}\sum_{j=1}^{N}\sum_{k=1}^{N}\sum_{l=1}^{N}a_{\mathrm{ijkl}}x_{i}x_{j}x_{k}x_{l}\\ +\sum_{i=1}^{N}\sum_{j=1}^{N}\sum_{k=1}^{N}\sum_{l=1}^{N}\sum_{m=1}^{N}a_{\mathrm{ijklm}}x_{i}x_{j}x_{k}x_{l}x_{m}+\cdots \end{array}$$

In this study, the dependent variable y is the normalized Raman intensity of the local mixture structure (indicated by *R*), and the independent variables $x_{1},x_{2},\ldots ,x_{N}$ correspond to the normalized Raman intensity of molecule *A* (shown by $r_{A}$) and molecule *B* (shown by $r_{B}$) and all fingerprints (shown by $r_{i}$, considering a total of *N* fingerprints) at every unique wavelength. We included the normalized Raman intensities of isolated molecules because in a practical scenario we would expect all the Raman intensity data of the mixtures to be contaminated by Raman intensities of isolated molecules.*^,^^†^

The unknown nonlinear correlation f between *R* and $r_{i}$ is given as $R=f\left(r_{A},r_{B},r_{1},r_{2},\ldots ,r_{N}\right)$. Eq. **2** gives the approximate function $\bar{f}$ that reproduces the normalized Raman spectra of the local mixture structure using fingerprint data, i.e., $\bar{R}=\bar{f}\left(r_{A},r_{B},r_{1},r_{2},\ldots ,r_{N}\right)$ (109), where a, $a_{i}$, $a_{ij}$, $a_{\mathrm{ijk}}$, and $a_{\mathrm{ijkl}}$ are all unknown constant coefficients.

[2] $$\begin{array}{l} \bar{R}=a+\sum_{i=A,B,1}^{N}a_{i}r_{i}+\sum_{i=A,B,1}^{N}\sum_{j=A,B,1}^{N}a_{ij}r_{i}r_{j}+\sum_{i=A,B,1}^{N}\sum_{j=A,B,1}^{N}\sum_{k=A,B,1}^{N}a_{\mathrm{ijk}}r_{i}r_{j}r_{k}+\cdots \\ \sum_{i=A,B,1}^{N}\sum_{j=A,B,1}^{N}\sum_{k=A,B,1}^{N}\sum_{l=A,B,1}^{N}a_{\mathrm{ijkl}}r_{i}r_{j}r_{k}r_{l}+\cdots \end{array}$$

The second term in Eq. **2** captures the direct contribution of each fingerprint to $\bar{R}$, the third term captures the pairwise contribution of overlapping/contamination for each pair of two fingerprints, the fourth term captures the pairwise contribution of overlapping/contamination of every three fingerprints, and so on.

Our previous work showed that truncating the fifth term and above in Eq. **2** had no significant effect on the accuracy of this kernel function in reproducing the Raman spectra. In fact, the coefficients $a_{ij}$ and $a_{\mathrm{ijk}}$ were included mostly to get a better fit, while $a_{i}$ directly reflected the relative strength of each fingerprint. Models using up to the fourth term in Eq. **2** produced desirable fits in the range of 0.5%. Therefore, the final form of our kernel function is given by Eq. **3**.

[3] $$\bar{R}=a+\sum_{i=A,B,1}^{N}a_{i}r_{i}+\sum_{i=A,B,1}^{N}\sum_{j=A,B,1}^{N}a_{ij}r_{i}r_{j}+\sum_{i=A,B,1}^{N}\sum_{j=A,B,1}^{N}\sum_{k=A,B,1}^{N}a_{\mathrm{ijk}}r_{i}r_{j}r_{k}\;.$$

### Application of Proposed Method to Generate the Computational Design Space.

The task of Eq. **3** is to extract the (intuitive) weights in a Raman signal. We defined the intuitive linear weights of each fingerprint (${a^{\prime }}_{i}$) based on the coefficients $a_{i}$ as ${a^{\prime }}_{i}=a_{i}/\left[\left[\sum_{i=A,B,1}^{N}a_{i}\right]-a_{A}-a_{B}\right]$, where i indicates the other N fingerprints. In this definition, the contributions to the spectra from *A* and *B* are removed and treated as noise.

Eq. **3** should be solved numerically to calculate the coefficients a, $a_{i}$, $a_{ij}$, and $a_{\mathrm{ijk}}$ and then the intuitive linear weights of each fingerprint ${a^{\prime }}_{i}$. For this purpose, we rewrote Eq. **3** in matrix form as R=ρA, where *R* is the vector of the normalized Raman intensity of the local mixture structure, *A* is a vector of all coefficients in Eq. **3**, and *ρ* is the matrix containing the normalized Raman intensities of all the fingerprints $r_{i}$. The Python code producing these matrixes and vectors is available in *SI Appendix*.

This system of equations, R=ρA, can be solved numerically as $A={\left(\rho^{T}\rho \right)}^{-1}\rho^{T}R$ (110), where the superscripts *^T^* and ^−1^ refer to matrix transposition and matrix inversion, respectively. The matrix *ρ* may become singular at certain numerical values of the computational normalized Raman intensities. Therefore, we computed its Moore–Penrose pseudoinverse using a least-squares solver (*linalg.pinv* in the NumPy package) (111).

### Design Space.

After applying the proposed method to all computed Raman intensities of the model mixtures, we used the results to construct the process design space $\left\{T,\tau ,t,a_{i}^{\prime }\right\}$. This design space was used to engineer the optimal operating condition for a target fingerprint represented by $a_{i}^{\prime }$. Note that τ and t can be correlated to each other through the design specification of the twin-screw granulator. This correlation is incorporated in the parameter *M* as $M=\psi \frac{tf\tau }{L}$, where *L* is the length of the twin-screw granulator, f is the forward carrying of material per rotation (screw lead), and ψ is a correction factor set as ψ=1.

### Implementation Proposal.

In real time, this design space accommodates a process controller that can manipulate any of the three operating parameters {T,τ,t} to target $a_{i}^{\prime }$ based on the actual Raman intensity signal from the spectrometer probe. In such a scenario, we would solve Eq. **3** for the Raman intensities measured by the probe as the vector *R*. This would result in a real-time calculation of the fraction of fingerprints. These calculated fractions would then be compared against the design space in Figs. 2–4, which acts as a decision tree for the controller to alter the screw rotation speed or temperature.

## Supplementary Material

Supplementary File

## Data Availability

Authors deposited all associated data in the Zenodo data repository, a publicly accessible database (112–116).

## Footnotes

*M. A. Khansary, G. M. Walker, S. Shirazian, “Correlating Raman spectra of ibuprofen, nicotinamide and their dimers” in Material Science and Engineering Congress (Deutsche Gesellschaft für Materialkunde e.V., Darmstadt, Germany, 2020).

^†^M. A. Khansary, G. M. Walker, S. Shirazian, “Analysis of Raman spectra signals based on molecular fingerprints from DFT data” in European Congress and Exhibition on Advanced Materials and Processes: EUROMAT 2021 (The Austrian Society for Metallurgy and Materials, Graz, Austria, 2021).
