# Supplementary material for: A molecularly enhanced proof of concept for targeting cocrystals at molecular scale in continuous pharmaceuticals cocrystallization
Source: Proc Natl Acad Sci U S A. 2022 May 20;119(21):e2114277119. doi: 10.1073/pnas.2114277119 (PMC9173768; doi:10.1073/pnas.2114277119)
Supplement: Supplementary File [file pnas.2114277119.sapp.pdf]

ID	Structure ↓	Normalized Raman spectra ↓
DI-1 →	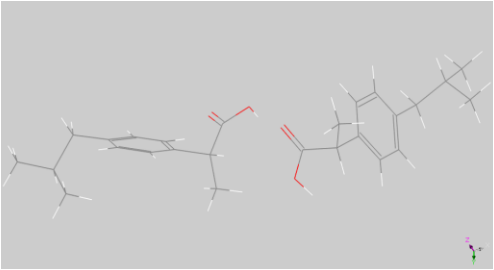	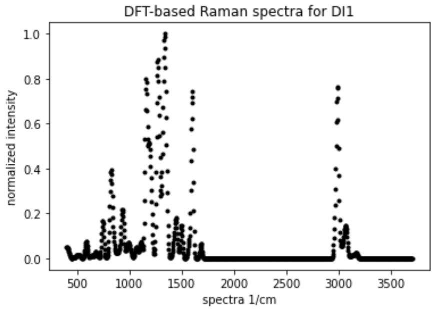
DI-2 →	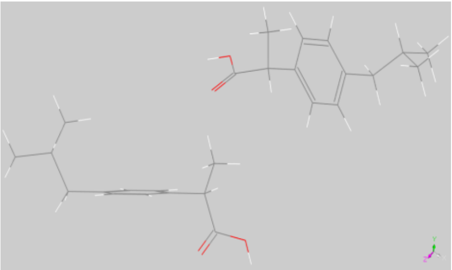	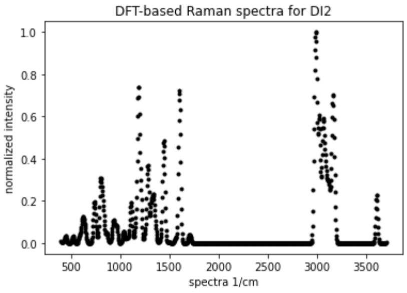
DI-3 →	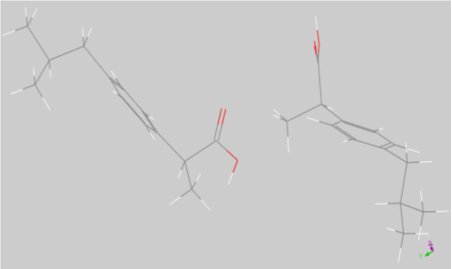	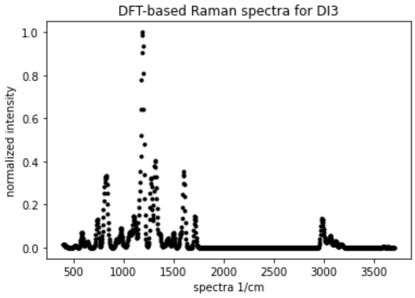
DN-1 →	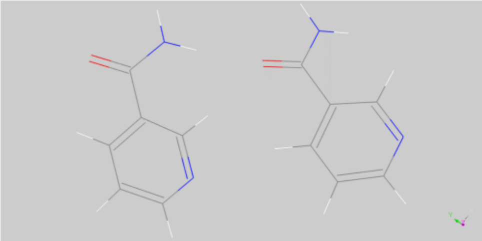	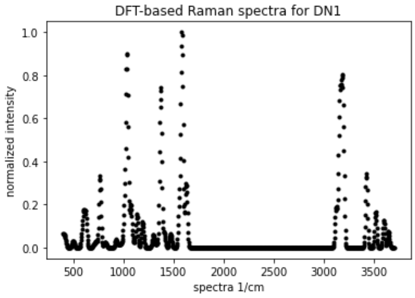
DN-2 →	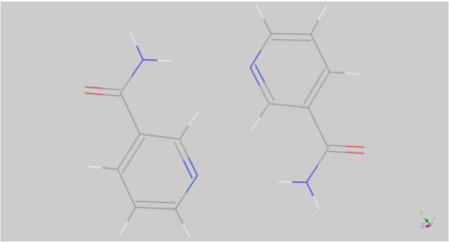	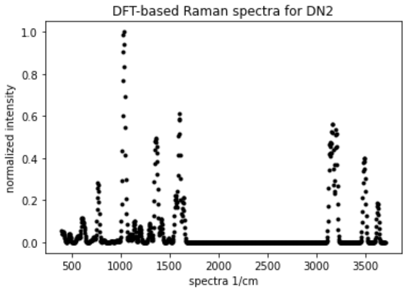

DN-3 →

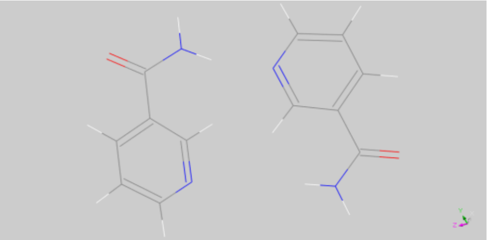

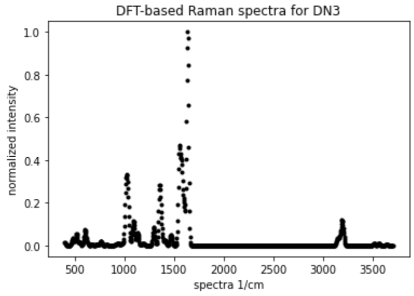

DN-4 →

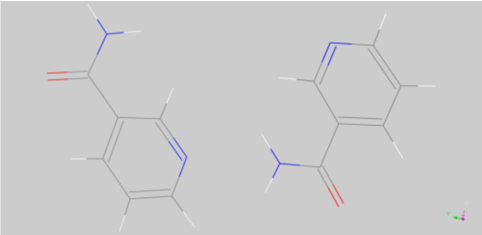

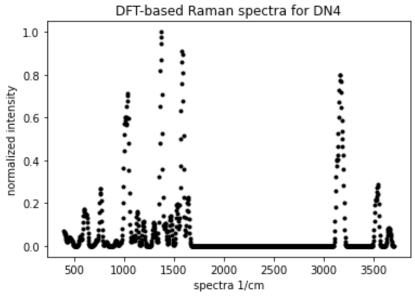

DN-5 →

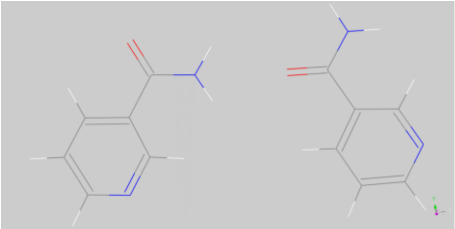

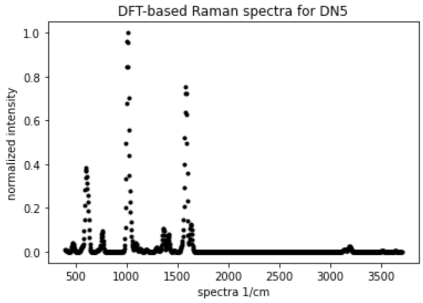

DN-6 →

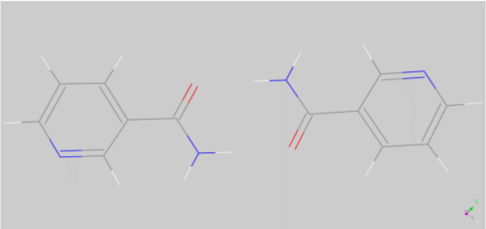

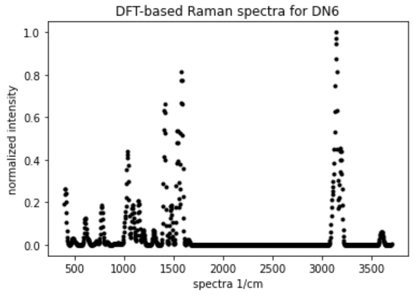

DN-7 →

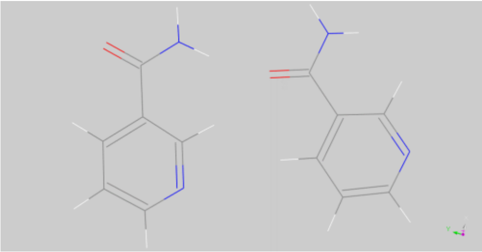

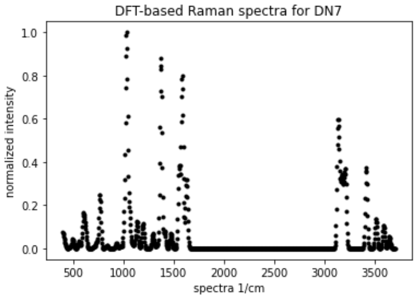

DN-8 →

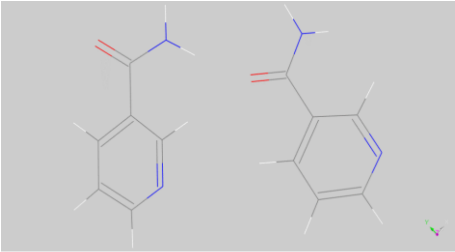

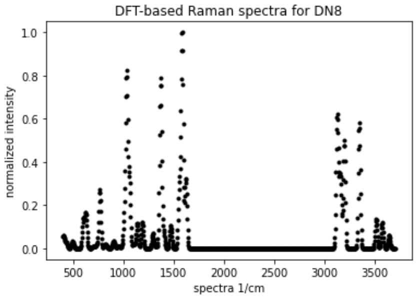

DN-9 →

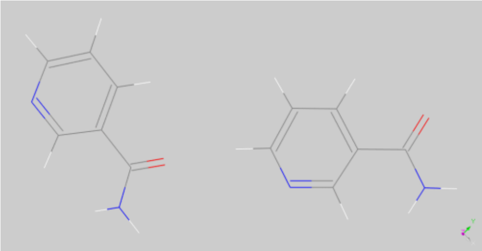

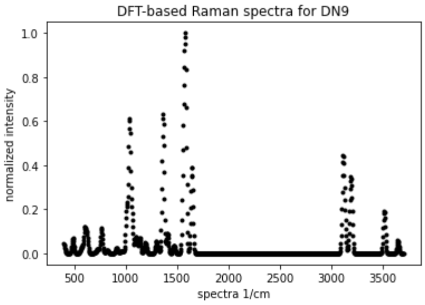

CO-1 →

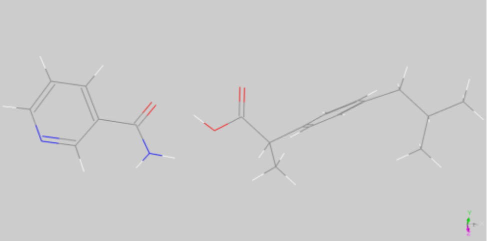

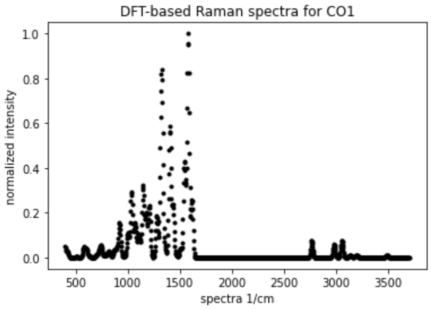

CO-2 →

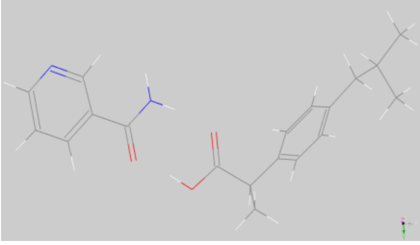

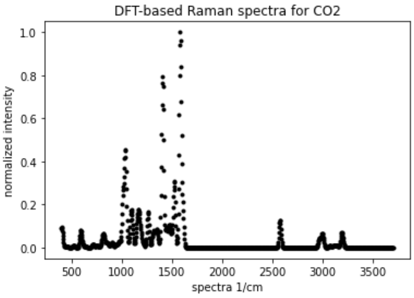

CO-3 →

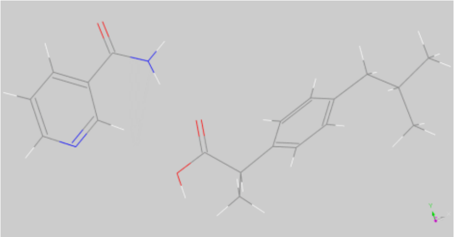

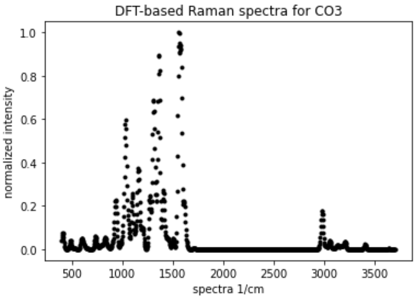

CO-4 →

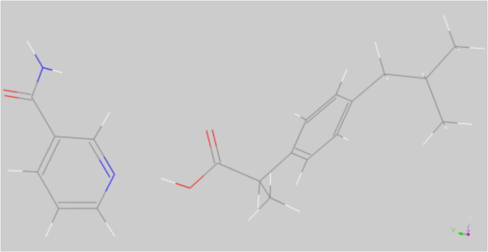

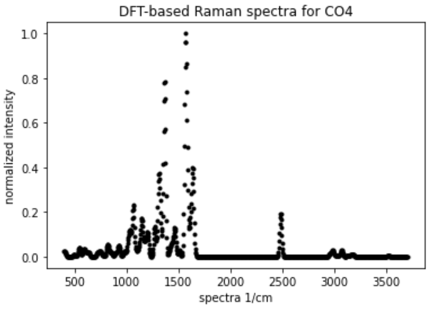

CO-5 →

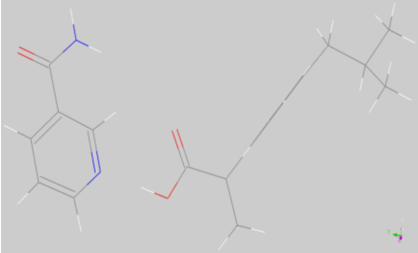

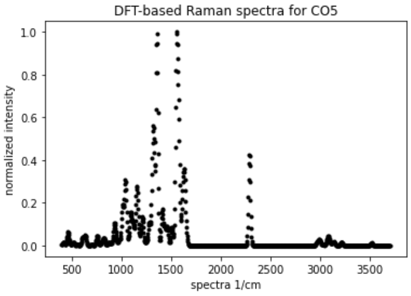

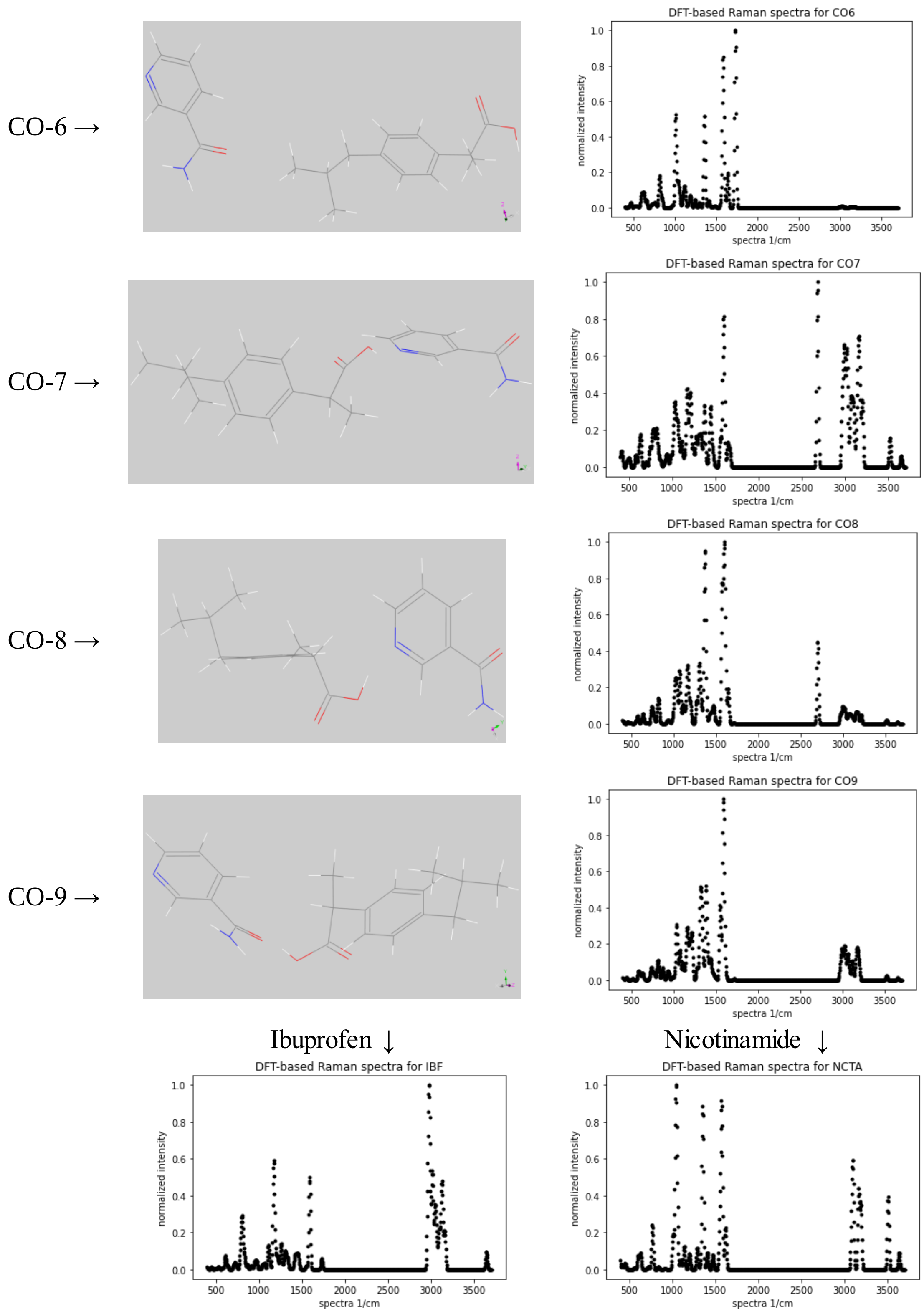

Figure S1. The fingerprints' structures and their computed Raman spectra [1-4].

References

1. Khansary, M.A., G. Walker, and S. Shirazian, *Incomplete cocrystalization of ibuprofen and nicotinamide and its interplay with formation of ibuprofen dimer and/or nicotinamide dimer: A thermo dynamic analysis based on DFT data*. International Journal of Pharmaceutics, 2020: p. 119992.

2. Khansary, M.A., G.M. Walker, and S. Shirazian. *Correlating Raman Spectra of Ibuprofen, Nicotinamide and their Dimers*. in *Material Science and Engineering Congress*. 2020. Darmstadt, Germany: Deutsche Gesellschaft für Materialkunde e.V.
3. Khansary, M.A., G.M. Walker, and S. Shirazian. *Analysis of Raman spectra signals based on molecular fingerprints from DFT data*. in *European Congress and Exhibition on Advanced Materials and Processes: EUROMAT 2021*. 2021. Graz, Austria: The Austrian Society for Metallurgy and Materials.
4. Asgarpour Khansary, M., S. Shirazian, and G. Walker, *Molecular engineering of cocrystallization process in holt melt extrusion based on kinetics of elementary molecular processes*. International Journal of Pharmaceutics, 2021. **601**: p. 120495.
